# Interpretation of ambiguity: Differences between children and adolescents with and without an anxiety disorder

**DOI:** 10.1016/j.jad.2015.08.022

**Published:** 2015-12-01

**Authors:** Polly Waite, Jon Codd, Cathy Creswell

**Affiliations:** a School of Psychology and Clinical Language Sciences, University of Reading, Reading RG6 6AL, UK; b Talking Therapies, Shinfield Medical Practice, Reading, UK

**Keywords:** Anxiety/anxiety disorders, Child/adolescent, Cognition

## Abstract

**Background:**

Theory and treatment of anxiety disorders in young people are commonly based on the premise that interpretation biases found in anxious adults are also found in children and adolescents. Although there is some evidence that this may be the case, studies have not typically taken age into account, which is surprising given the normative changes in cognition that occur throughout childhood. The aim of the current study was to identify whether associations between anxiety disorder status and interpretation biases differed in children and adolescents.

**Methods:**

The responses of children (7–10 years) and adolescents (13–16 years) with and without anxiety disorders (*n*=120) were compared on an ambiguous scenarios task.

**Results:**

Children and adolescents with an anxiety disorder showed significantly higher levels of threat interpretation and avoidant strategies than non-anxious children and adolescents. However, age significantly moderated the effect of anxiety disorder status on interpretation of ambiguity, in that adolescents with anxiety disorders showed significantly higher levels of threat interpretation and associated negative emotion than non-anxious adolescents, but a similar relationship was not observed among children.

**Conclusions:**

The findings suggest that theoretical accounts of interpretation biases in anxiety disorders in children and adolescents should distinguish between different developmental periods. For both ages, treatment that targets behavioral avoidance appears warranted. However, while adolescents are likely to benefit from treatment that addresses interpretation biases, there may be limited benefit for children under the age of ten.

## Highlights

• Cognitive theories of anxiety emphasize the role of interpretation bias.
• We examine interpretation bias in anxious and non-anxious children and adolescents.
• Anxious adolescents show more threat interpretation than non-anxious adolescents.
• Anxious and non-anxious children show similar levels of threat interpretation.
• Theories should distinguish between different developmental periods.

## 1. Introduction

Anxiety disorders are highly prevalent among children and adolescents and have far-reaching negative consequences (Essau and Gabbidon, 2013). A central tenet of cognitive theories of anxiety in adults is the idea that anxious individuals are inclined to excessively infer future threat/danger in their environment and to underestimate their ability to cope, and this leads to physiological arousal and behavioral avoidance, thus maintaining anxiety (Beck and Clark, 1997). This is supported by studies demonstrating that adults with elevated anxiety show attentional biases towards threatening stimuli (MacLeod et al., 1986, Mogg et al., 1992) and a tendency to interpret ambiguous information in a disproportionally threatening way (Amir et al., 2005, Mathews and Mackintosh, 2000). Accordingly, Cognitive Behavior Therapy (CBT) targets these cognitive processes so that, for example, the individual is able to challenge his or her biased cognitions to think in a more benign way. This approach has been extended to young people with anxiety disorders, based on the assumption that the information-processing biases found in adults are also found in children and adolescents.

Over the past 20 years, evidence has accumulated to suggest that there is an association between anxiety diagnoses or symptoms and threat-related interpretation biases in children and young people across relatively broad age ranges (ranging from 7 to 18 years) (e.g., Barrett et al., 1996; Creswell et al., 2005). There have been some inconsistencies in findings, however, particularly among studies with children at the younger end of this age range (7–12 years). While some studies have continued to find significant group differences around interpretation of threat/danger on tasks involving ambiguous scenarios (e.g., Alkozei et al., 2014; Waters et al., 2008b), other studies have failed to find differences in judgments of threat between children with anxiety disorders and non-anxious children of the same age on similar tasks (Creswell et al., 2014, Waters et al., 2008a) or a homographs task (Waters, et al., 2008b).

Theoretical accounts of anxiety suggest that once threat is detected in the environment, dedicated neural circuitry then increases physiological arousal and inhibits ongoing behavior to deal with the threat (Gray and McNaughton, 2003, Öhman and Mineka, 2001). Physiological arousal increases as a result of the relatively strong link between the cognitive representations of emotional states and mood congruent events. Behavioral avoidance of anxiety-producing stimuli then maintains the anxiety because it interferes with the individual’s ability to experience a threatening or emotional event in a more benign way (Foa and Kozak, 1986, Mowrer, 1960). As such, as well as examining whether children and young people make more threatening interpretations of ambiguous stimuli, studies have investigated levels of associated negative emotion, perceptions of coping and choice of behavioral strategies. For example, Waters et al. (2008a) found significant associations between anxiety disorder status and anticipated negative emotion in children aged 7–12 years. In addition, three studies have found that children and young people with anxiety disorders are significantly more likely than non-anxious children to underestimate their ability to control or influence the outcome of the situation (Bögels and Zigterman, 2000, Creswell et al., 2014, Waters et al., 2008a), although it is of note that Creswell et al. (2014) only found a significant difference amongst those aged 10–12 years and not those aged 7–9 years. Finally, there are also mixed findings in relation to predicted behavioral responses to potentially threatening situations. Whereas Chorpita et al. (1996) found a significant association between anxiety symptoms and avoidant plans of action amongst children aged 9–13 years of age, neither Barrett et al. (1996) nor Bögels et al. (2003) found a significant association among children, aged 7–14 years and 7–12 years respectively. All three studies used ambiguous scenarios and therefore differences in findings may relate to participant characteristics, with participants in Chorpita et al.’s study being older (mean age of 11.3 years) than participants in the other two studies (mean ages ranged from 9.0 to 10.2 years). However, the small sample size in Chorpita et al.’s study (anxious group *n*=4; non-anxious group *n*=8) limits interpretation of the findings.

As is evident from the studies described above, the most widely used measure of interpretation bias is an ambiguous story paradigm, involving the verbal presentation of hypothetical situations that could be interpreted as threatening or non-threatening. This paradigm was originally used with adults (Butler and Mathews, 1983), and then subsequently modified for use with children (Barrett et al., 1996, Chorpita et al., 1996) as young as five years of age (Creswell et al., 2011). Studies differ in the content of the scenarios described (e.g. social and physical threat, threat relating to different anxiety disorders, inclusion of information about physical symptoms or degree of ambiguity or threat), length of scenarios (ranging from one sentence to a number of sentences), the number of scenarios (ranging from three to 12), the wording of the questions and types of response (e.g. free or forced choice). However, when considering these methodological differences, no clear patterns emerge that explain the inconsistent findings between studies, suggesting that other factors (such as participant age) may be of greater relevance.

Although studies have often included both children and adolescents across broad age ranges (Bögels and Zigterman, 2000, Taghavi et al., 2000), the extensive literature on cognitive development suggests that there are key differences between children and adolescents. The adolescent years are characterized by the maturation of cognitive and emotional abilities (Yurgelun-Todd, 2007), in line with prefrontal neurological development (Gogtay et al., 2004). Developmental theories emphasize greater capacity for abstract, hypothetical reasoning in adolescents compared to children (Piaget and Inhelder, 1969), and increasing attentional capacity, processing speed, decision-making, ability to selectively attend to information, regulate emotion, inhibit responses and control behavior that continues throughout the adolescent period (Adleman et al., 2002, Anderson et al., 2001, Hooper et al., 2004, Luna et al., 2004). There is also some suggestion that there may be differences in the nature of the association between thinking styles and affect between childhood and adolescence. Specifically there is evidence that in middle childhood, events rather than explanatory style, predict high levels of negative affect, whereas by early adolescence, explanatory style on its own or in conjunction with life events becomes a significant predictor of affect (Nolen-Hoeksema et al., 1992). This suggests that cognitive accounts of disordered affect may begin to apply in adolescence, rather than in childhood.

Given the normative changes to cognition throughout childhood and adolescence, it is striking that age has not typically been taken into account in studies of interpretation bias and anxiety. Only one study has examined associations between interpretation biases and anxiety in adolescents specifically, finding that adolescents from a community population aged 11–16 years with a high level of social anxiety symptoms had significantly higher levels of threat interpretation than those with low social anxiety symptoms (Miers et al., 2008). To date, no studies have examined interpretation biases exclusively in adolescents with anxiety disorders or contrasted adolescents with younger age groups.

Further research is needed to examine interpretation biases in the context of anxiety disorders with age groups that correspond to distinct developmental stages. As such, the current study examined the hypothesis that children and adolescents with anxiety disorders will exhibit significantly higher levels of threat interpretation, anticipated negative emotion, predicted avoidant behaviors and lower levels of perceived control in response to ambiguity than non-anxious children and adolescents. We also set out to explore whether differences between anxious and non-anxious groups were stronger for adolescents compared to children, i.e., whether age group moderated the association between anxiety disorder status and threat interpretation.

We chose to use an ambiguous scenarios paradigm to measure interpretation bias because it is the most widely used measure of interpretation of ambiguity in relation to anxiety in children and young people, and therefore allows us to draw meaningful comparisons with existing studies, and is less reliant on knowledge of specific vocabulary than, for example, a homograph-based task (Waters et al., 2008b).

## 2 Methods

### 2.1 Participants

#### 2.1.1 Children and adolescents with anxiety disorders

All participants with anxiety disorders were referred by primary and secondary care services for treatment of an anxiety disorder. To be included in the study, all children/adolescents were required to meet diagnostic criteria for a current anxiety disorder on the Anxiety Disorders Interview Schedule (ADIS-C/P; Silverman and Albano, 1996) and for this to be identified as the primary problem. They were not invited to participate if they had an autistic spectrum disorder, significant intellectual impairment, a risk of deliberate self-harm, if they were currently receiving therapy, or if they did not understand and speak English. No participants in the study were taking psychoactive medication.

Thirty adolescents aged between 13 and 16 years, who met diagnostic criteria for an anxiety disorder, were recruited. We then selected 30 children aged 7–10 years, who had been diagnosed with an anxiety disorder and had completed the same assessment as part of a wider study; the data from these participants has not been published elsewhere. The children with anxiety disorders were selected to match the adolescent group on their primary anxiety disorder, comorbid mood and behavior disorders, gender, ethnicity and socio-economic status. Table 1 provides information on the sample characteristics for all participants. For both groups, the primary anxiety disorder diagnoses were: social anxiety disorder (27%), specific phobia (30%), generalized anxiety disorder (23%), panic disorder, with or without agoraphobia (17%) and agoraphobia without panic disorder (3%).

#### 2.1.2 Non-anxious children and adolescents

Thirty non-anxious adolescents aged 13–16 years were recruited. A further 30 non-anxious children aged 7–10 years were selected from a wider study in order to match the children/adolescent groups where possible on demographic variables.^1^ All non-anxious participants were recruited through advertisements sent to local schools and youth groups. To be included in the study, all non-anxious participants were required to score below clinical cut-offs on the SCAS-P and the SMFQ-P, speak English and have no significant intellectual impairment.

### 2.2 Measures

#### 2.2.1 Diagnoses

Children and adolescents' diagnoses were determined using the ADIS-C/P (Silverman and Albano, 1996). This structured interview, designed to assess current DSM-IV anxiety disorders, as well as current mood and behavioral disorders, has good psychometric properties (Silverman et al., 2001). As is standard, if the child/adolescent met symptom criteria for a diagnosis, on the basis of his/her report or that of his/her parent, the assessor assigned a Clinician Severity Rating (CSR), ranging from 0 (*absent* or *none*) to 8 (*very severely disturbing/disabling*); a CSR of 4 or more indicated the child/adolescent met criteria for diagnosis. The diagnosis with the highest CSR was classed as the primary diagnosis. Overall inter-rater reliability for the assessment team was good to excellent: child report, *M*=0.97 (range 0.88–1.00), parent report, *M*=0.98 (range 0.92–1.00) and for CSR scores was: child report, *M*=0.98 (range 0.91–1.00) and parent report, *M*=0.98 (range 0.96–1.00).

#### 2.2.2 Symptoms

The Spence Children's Anxiety Scale (SCAS-C/P; Spence, 1998) assesses child/adolescent and parent-reported symptoms. The SCAS includes 38 items to assess anxiety symptoms (and 6 positive filler items in the child version), each scored on a 4-point scale, ranging from 0 (*never*) to 3 (*always*). The measure has been validated for use with children/adolescents aged 6–18 years and both versions have good reliability, as well as discriminant and convergent validity (Nauta et al., 2004, Spence et al., 2003). Internal consistency for these scales was excellent (SCAS-C *α*=0.92; SCAS-P *α*=0.94).

The Short Mood and Feelings Questionnaire (SMFQ-C/P; Angold et al., 1995) is a self-report measure to assess child/adolescent depression. The child/adolescent and parent versions both have 13 items, each scored on a 3-point scale (‘*not true*’, ‘*sometimes*’ or ‘*true*’). The scale has been validated with children/adolescents aged 6–17 years and has good internal reliability and discriminant validity (Angold et al., 1995). Internal consistency for the SMFQ was good to excellent (SMFQ-C *α*=0.86; SMFQ-P *α*=0.93).

#### 2.2.3 Ambiguous scenarios

The Ambiguous Scenarios Questionnaire (ASQ) (Barrett et al. (1996) was originally developed for administration with children and young people aged 7–14 years, and consists of 12 hypothetical situations (six social and six non-social). We used a modified version (Creswell et al., 2014) in which, after being presented with each ambiguous scenario (e.g., ‘You see the head teacher walking around the school grounds and they have been asking other students/children where you are’), participants are asked to (a) rate how they would feel in this situation (0=*not at all upset*; 10=*very upset; negative emotion*), (b) give a free response to the question ‘Why do you think this is happening?’ (threat free response), (c) rate how much they would be able to do about this situation (0=*nothing*, 10=*a lot*) (perceived control), (d) give a free response to the question ‘What would you do?’ (behavior free response) and, (e) choose which of two alternatives (threat/non-threat) they would be more likely to think in this situation (threat forced choice) (e.g., ‘The head teacher thinks you have done something wrong’ or ‘The head teacher has a message from your parent for you’).

Free responses were coded as threat/non-threat and avoidance/non-avoidance, by a psychology undergraduate, blind to participant group and scores on all other measures. A second independent coder (undergraduate psychologist) coded a sample of the responses (*n*=26) in order to assess inter-rater reliability. Intraclass correlations were good, ICC for threat was 0.97 and ICC for avoidance was 0.75. Scores for each domain were totaled across the 12 scenarios. Internal consistency was excellent for negative emotions (children *α*=0.82, adolescents *α*=0.91), good for threat (children *α*=0.82, adolescents *α*=0.91) and acceptable for control (children *α*=0.78, adolescents *α*=0.88). The poor internal consistency for avoidance (children *α*=0.28, adolescents *α*=0.40) is likely to reflect the low frequency of avoidant behavior within both child and adolescent samples.

### 2.3 Procedure

Ethical approval for the study was given by the National Research Ethics Service (NRES) London-Brent Research Ethics Committee and the University of Reading Ethics Committee. All participants provided informed consent after the nature of the procedures was explained, prior to taking part in the research.

The children and adolescents with anxiety disorders and their parents were seen for an initial assessment, to complete standardized questionnaires and undertake the diagnostic interview, carried out by psychology graduates who received thorough training and regular supervision. If the child/adolescent met the inclusion criteria for the study, the study was discussed with them and their parent, and they were given the information sheet and consent form to take away and read. For the non-anxious participants, if they expressed an interest in the study, they were sent consent forms, information sheets and the screening measures to complete and return. Eligible and consenting participants then completed a laboratory-based assessment at the university, which included the ASQ. The ASQ was administered verbally, audio-recorded and the researcher wrote down the participants' answers.

## 3 Results

### 3.1 Data reduction, analytic strategy and preliminary analyses

Continuous data was screened to examine whether it met assumptions of normality and, with the exception of the domain of perceived control, assumptions were violated. Attempts to transform the data were unsuccessful and therefore, analyses were run parametrically with 1000 bootstrap samples. All tests were two-tailed.

We began by conducting bivariate correlations to establish the extent of the association between ASQ responses. As in previous reports (e.g., Creswell and O’Connor, 2006), the free and forced choice threat responses correlated highly (*r*=0.61) and therefore were combined to reduce the number of variables. Although there were also significant correlations (at *p*<0.01) between negative emotions and control (*r*=0.33), negative emotions and threat (*r*=0.53), and threat and avoidance (*r*=0.44), these domains were analyzed separately as we were interested in their distinct roles.

To address the hypotheses, multivariate analyses of variance (MANOVA), using Pillai's trace, were carried out, with anxiety (anxiety disorder or non-anxious), age group (child or adolescent) and their interaction entered as independent variables. Threat interpretation, negative emotions, control and avoidance were entered as dependent variables. Where the effects of the interaction were significant, *t*-tests were used to explore differences between groups. Group means are presented in Table 2. Although the clinically anxious groups were matched for mood disorder diagnoses, we also conducted the analyses controlling for depressive symptoms, with scores on the SMFQ-C/P as a covariate, and also repeated the analyses excluding the five children and five adolescents with comorbid mood disorders. Results were broadly consistent but where there was a difference in findings, this is highlighted. Finally, because there were group differences on SES (Table 1), further sensitivity analyses were undertaken using MANCOVA, controlling for SES and as this did not change the results, analyses are reported without the inclusion of SES. We also examined gender, both as a covariate and a moderator of the effect of anxiety status, and found no significant main effect of gender, no difference in the overall pattern of results when controlling for gender and no significant gender×anxiety group interaction effects.

### 3.2 Hypothesis testing

The results of the MANOVA indicated that there was a significant effect of anxiety disorder (*V*=0.11, *F*[4,112]=3.34, *p*=0.01) and age group (*V*=0.11, *F*[4,112]=3.46, *p*=0.01) and a significant anxiety disorder by age group interaction (*V*=0.16, *F*[4,112]=5.25, *p*=0.001) on participants' responses.

While this same pattern was observed when we excluded participants with comorbid mood disorders from the analysis, when SMFQ scores were entered as a covariate, the significant main effect of anxiety disorder was no longer significant for child/adolescent report (*V*=0.06, *F*[4,108]=1.80, *p*=0.13) and parent report (*V*=0.03, *F*[4,110]=0.83, *p*=0.51), although the significant effect of age group (SMFQ-C: *V*=0.10, *F*[4,108]=3.05, *p*<0.01; SMFQ-P: *V*=0.12, *F*[4,110]=3.58, *p*<0.01), and anxiety disorder by age group interaction maintained (SMFQ-C: *V*=0.13, *F*[4,108]=3.90, *p*<0.01; SMFQ-P: *V*=0.14, *F*[4,110]=4.62, *p*<0.01).

**Fig. 1:**
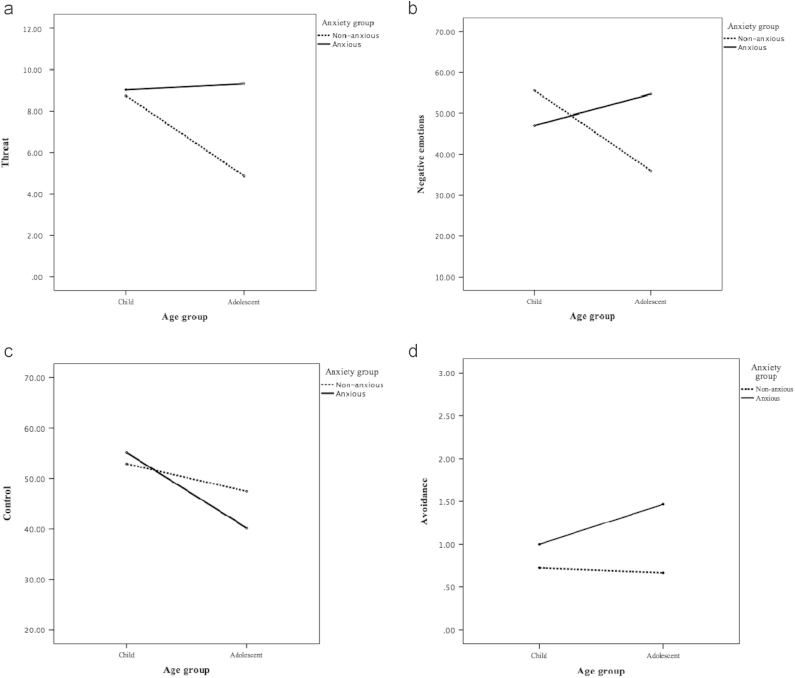
Interactions between anxiety disorder and age group. (a) Threat, (b) negative emotions, (c) control, and (d) avoidance.

There was a significant effect of anxiety disorder for threat interpretation (*F*[1,115]=10.60, *p*=0.001, *ω*²=0.07), with significantly more threat responses given by children and adolescents with an anxiety disorder (mean=9.18, *SD*=4.46), compared to non-anxious children and adolescents (mean=6.80, *SD*=3.96). There was also a significant effect of age group (*F*[1,115]=5.68, *p*=0.02, *ω*²=0.03), with significantly more threat responses given by children (mean=8.88, *SD*=4.04), compared to adolescents (mean=7.10, *SD*=4.52). As shown in Fig. 1a, the interaction between age and anxiety group was also statistically significant (*F*[1,115]=7.79, *p*=0.01, *ω*²=0.05) with adolescents, but not children, with an anxiety disorder showing significantly more threat interpretation compared to their non-anxious counterparts (*t*(58)=4.37, *p*<0.01, *d*=1.13; *t*(58)=0.29, *p*=0.78, *d*=0.08 respectively). A significant main effect of anxiety disorder was also observed both when we excluded participants with a comorbid mood disorder and when we entered child/adolescent reported SMFQ scores; however it became non-significant when parent-reported SMFQ scores were entered as a covariate (*F*[1,113]=2.08, *p*=0.15, *ω*²=0.01).

For anticipated negative emotions, neither the effect of anxiety disorder (*F*[1,115]=1.69, *p*=0.20, *ω*²=0.01), nor the effect of age group was significant (*F*[1,115]=2.51, *p*=0.12, *ω*²=0.01). However, the interaction between age and anxiety group was statistically significant (*F*[1,115]=12.99, *p*=<0.001, *ω*²=0.09). As shown in Fig. 1b, adolescents (but not children) with an anxiety disorder anticipated significantly more negative emotions, compared to their non-anxious counterparts (*t*(58)=3.17, *p*<0.01, *d*=0.83; *t*(58)=−1.81, *p*=0.08, *d*=0.47 respectively).

For perceived control the effect of anxiety disorder was not significant, (*F*[1,115]=0.37, *p*=0.54, *ω*²=0.01), however the effect of age group was significant (*F*[1,115]=6.00, *p*=0.02, *ω*²=0.04), with evidence of lower coping expectations among adolescents (mean=43.80, *SD*=24.59), compared to children (mean=53.97, *SD*=20.20). As can be seen in Fig. 1c, the interaction between age and anxiety group was not statistically significant (*F*[1,115]=1.30, *p*=0.26, *ω*²<0.001).

For avoidance there was a significant effect of anxiety disorder (*F*[1,115]=7.86, *p*=0.01, *ω*²=0.05), with significantly more avoidant responses given by children and adolescents with an anxiety disorder (mean=1.23, *SD*=1.23), compared to non-clinical children and adolescents (mean=0.69, *SD*=0.84). However, neither the effect of age group (*F*[1,115]=1.14, *p*=0.29, *ω*²<0.001), nor the interaction between age and anxiety group (*F*[1,115]=1.87, *p*=0.18, *ω*²=0.01) (Fig. 1d) was statistically significant. Consistent with the finding for threat interpretation, while the significant main effect of anxiety disorder was also observed when we excluded participants with a comorbid mood disorder from the analysis and when we entered child/adolescent-reported SMFQ scores, it became non-significant when parent-reported SMFQ scores were entered as a covariate (*F*[1,113]=1.78, *p*=0.19, *ω*²=0.01).

To summarize, compared to non-anxious children and adolescents, children and adolescents with anxiety disorders exhibited significantly higher levels of threat interpretation and predicted avoidant behaviors, but there were no significant differences related to anticipated negative emotion or perceptions of control. Adolescents reported significantly lower levels of threat interpretation and coping expectations compared to children but there were no significant differences between the age groups for negative emotion or perceived control. Of particular note, we found that age group moderated the association between anxiety disorder status and threat interpretation with adolescents, but not children, with an anxiety disorder showing significantly more threat interpretation and predicting more negative emotion compared to their non-anxious counterparts.

## 4 Discussion

The aim of this study was to examine interpretation biases in children and young people in distinct developmental stages (middle childhood and adolescence) with and without anxiety disorders. We found that adolescents with an anxiety disorder showed significantly more threat interpretation and anticipated more negative emotion, compared to non-anxious adolescents, whereas a similar relationship was not observed among the two child groups. This remained the case when mood disorders, depressive symptoms and socio-economic status were taken into account. As hypothesized, significantly more avoidant responses were given by both children and adolescents with an anxiety disorder, compared to non-anxious children and adolescents. Contrary to our hypotheses, we did not find an effect of anxiety disorder for negative emotions or perceptions of control.

The finding that, compared to non-anxious adolescents, adolescents with anxiety disorders show significantly higher levels of threat interpretation and anticipated negative emotion is consistent with the one existing community-based study with a similar age group (Miers et al., 2008), and studies involving adults (e.g., Amir et al., 2005; Mathews and Mackintosh, 2000). It is also consistent with some preliminary studies of cognitive bias modification of interpretation (CBM-I), that have shown changes in anxiety when biases are modified in non-anxious adolescents (Lau et al., 2013, Telman et al., 2013) and clinically anxious adolescents (Reuland and Teachman, 2014) (although see Salemink and Wiers (2011) for conflicting findings). Together, these findings provide support for the notion that treatment focused on addressing interpretation biases is warranted among adolescents with anxiety disorders (e.g., Micco et al., 2007).

The lack of a significant difference between the children with anxiety disorders and non-anxious children for threat interpretation is consistent with the findings of Creswell et al. (2014) and Waters et al. (2008a), but discrepant with findings from other studies (e.g., Alkozei et al., 2014; Waters et al., 2008b). Furthermore in contrast to Creswell et al. (2014) and Waters et al. (2008a), we failed to find significant differences between anxious and non-anxious children on anticipated negative emotion. There were also no significant differences between these groups for perceptions of coping, which was consistent with Creswell et al. (2014), where a significant difference was only found for 10–12 year old and not 7–9 year old children, but not with studies involving children of broader age ranges, e.g., Bögels et al. (2003) and Waters et al. (2008a). The lack of significant associations between interpretation of ambiguity and anxiety among children is in line with CBM-I studies in which changes in threat interpretation have not consistently translated in to changes in anxiety (e.g., Lester et al., 2011; Vassilopoulos et al., 2013). Although studies suggest that a cognitive element is associated with treatment gains in children (e.g., Kendall and Treadwell, 2007; Peris et al., 2014), they typically involve children across broader ages than the current study (e.g., 9–13 years; 7–12 years, respectively). Indeed, inconsistencies in the literature to date may reflect differences in the distribution of child age within the broad age categories included in these studies.

Our findings suggest that although children with anxiety disorders aged 7–10 years show similar levels of threat interpretation and anticipated negative emotion to adolescents with anxiety disorders, this is also the case for non-anxious children. It is likely that for non-anxious children, at some point generally after the age of 10 years, they are able to inhibit these biases, perhaps as their thinking styles become more stable and as they develop a greater body of experiences to draw from to inform their thinking (e.g., Nolen-Hoeksema et al., 1992).

Unexpectedly, we found that perceptions of coping were related to age and not anxiety status, with significantly lower levels of coping expectations among adolescents compared to children. This may seem counter-intuitive given the research suggesting that locus of control becomes more internal over time, especially around mid-adolescence (Chubb et al., 1997) and that adolescents are likely to have a wider repertoire of skills to draw on than children when dealing with ambiguous situations. Instead, however, this finding may reflect adolescents having more experience in their lives of not feeling in control, such as within the school environment and social relationships and a greater awareness than children of the limits of their abilities to deal with certain situations. Similarly they may feel constrained in what they can do, especially in ambiguous social situations, due to a desire to fit in and be accepted by peers. In contrast to the findings for threat interpretation and negative emotion, there was neither a significant main effect of anxiety nor a significant interaction between age and anxiety. However the (non-significant) pattern of results is consistent with a pattern of reduced perceived control among anxious adolescents specifically. Further studies, powered to detect smaller effects, will be useful to explore this further.

Although there were relatively low levels of anticipated avoidance across all groups, children and adolescents with anxiety disorders were significantly more likely to suggest the use of a strategy involving avoidance than their non-anxious counterparts. This is accordant with the cognitive behavioral model of anxiety in adults, where avoidance is understood to prolong anxiety by impeding new learning and supports the inclusion of strategies to overcome avoidance in treatment for children and adolescents with anxiety disorders. Notably this finding is consistent with Chorpita et al. (1996), where the mean age of participants was older than the two studies (Barrett et al., 1996, Bögels et al., 2003) that did not find an association between child anxiety and avoidance. Although the effect of anxiety disorder status on avoidant behaviors remained significant when child-reported symptoms of mood were controlled for, it became non-significant when parent-reported child/adolescent symptoms of low mood were included as a covariate. This may reflect the fact that avoidance is associated with symptoms of both anxiety and depression, or perhaps that there is considerable shared variance in parent-reported anxiety and depression. Unlike threat interpretation and negative emotion, there was not a moderating effect of age. There were, however, elevated levels of anticipated avoidance among the anxious adolescents compared to the anxious children, suggesting that avoidance is increasingly used, albeit unsuccessfully, as a means of trying to deal with feared situations. The lack of increase in anticipated avoidance with age in the non-anxious group is consistent with a community-based study by Miers et al. (2014), who found that at the age of nine, youngsters who went on to show either low or high levels of avoidance of social situations in adolescence were hardly distinguishable.

The results of this study should be considered in light of the limitations. The cross-sectional nature of the study design means that conclusions cannot be drawn with regards to the direction of effects (i.e. whether interpretations biases have a causal influence on anxiety). On the basis that childhood and adolescence can be seen as distinct, developmental periods (Erikson, 1968), we considered age within two categories, but of course, changes are unlikely to occur in such a discrete way. The mean SCAS-C score for the children with anxiety disorders was lower than would be expected on the basis of the published norms, but is in line with other clinical studies (e.g., Hudson et al., 2009). This may reflect a lack of ability for children with anxiety disorders to reflect upon and accurately report their own internal state at this age, difficulty fully understanding what is meant by some questions, a desire to please by minimizing the problem, or discomfort in disclosing information (Kendall and Chansky, 1991, Ronan, 1996). Finally, we were underpowered to examine whether there were anxiety-disorder specific associations and this would be an important direction for further research.

The finding that, compared to non-anxious adolescents, adolescents with anxiety disorders had significantly higher levels of threat interpretation and negative emotion suggests that the adult cognitive model of anxiety (Beck and Clark, 1997) may be equally applicable to adolescents. However, the lack of significant differences between anxious and non-anxious children fails to support the validity of the model for children under the age of ten years and inevitably leads to the question of whether cognitive strategies are required in interventions for anxiety disorders in middle childhood. We cannot determine from the current study whether the lack of significant findings for children relate to methodological factors, for example difficulties in accurately reflecting on how one would respond to hypothetical situations, particularly in the absence of elevated affect. However the methods used here are not dissimilar to methods commonly used in generic CBT approaches to childhood anxiety disorders using thought records to examine evidence for and against negative thoughts (e.g., Kendall and Hedtke, 2006; Rapee et al., 2006). At the very least, the current findings suggest that attempting to challenge thoughts in this way may not be indicated with children less than 10 years of age.

The development of more ecologically valid measures of interpretation of ambiguity will be required to test to what extent the findings are influenced by differences in how children and adolescents respond to hypothetical scenarios. Indeed, the findings may also reflect the possibility that the relative contribution of cognitive factors and other factors (such as biological vulnerability, life events/lifestyle factors and learning through the behavior of parents and other key people in the young person's life; Murray et al., 2009) vary substantially throughout development and cognitions may make an increasingly influential contribution to changes in affect throughout adolescence (e.g., Nolen-Hoeksema et al., 1992). A clearer developmentally informed understanding of factors that maintain anxiety disorders in childhood and adolescence is required in order to inform and improve interventions for these common and debilitating disorders.

## 5 Conclusion

To conclude, the findings from the current study are consistent with the suggestion that key aspects of adult cognitive models of anxiety are applicable to adolescents and that treatments focused on addressing interpretation biases and avoidance are warranted. Compared to their non-anxious peers, children with anxiety disorders under the age of 10 years did not show significantly greater threat interpretations, negative emotion or reduced expectations around coping. There were, however, higher levels of avoidance amongst children and adolescents with anxiety disorders, compared to those without, which is consistent with the view that, for both age groups, behavioral strategies addressing avoidance should be an important part of treatment.

## Figures and Tables

**Table 1:** Sample characteristics.

	**Anxious children (*n*=30)**	**Non-anxious children (*n*=30)**	**Anxious adolescents (*n*=30)**	**Non-anxious adolescents (*n*=30)**	**Statistics**
**Child/adolescent gender (boys:girls)**	14:16	20:10	14:16	16:14	*χ*²(3)=3.21, *p*=.36
**Age in months (mean,*****SD*****, range)**	112.20 (10.49), 94–130^a^	110.60 (9.77), 96–131	181.50 (13.48), 158–198^a^	183.03 (13.79), 161–205	*F*(3,116)=348.21, *p<0*.001
**Ethnicity (% White British)**	93%	93%	93%	90%	*χ*²(3)=0.36, *p*=0.95
**Family SES (% “higher” or “professional”)**	67%	73%	67%^a^	97%^a^	*χ*²(3)=10.01, *p*=0.02
**SCAS-c total (mean,*****SD*****, range)**	36.20 (19.03), 10–81	27.89 (10.74), 6–44	39.23 (17.62), 10–87^b^	10.97 (5.54), 2–24^b^	*F*(3,111)=22.30, *p<*0.001
**SCAS-p total (mean,*****SD*****, range)**	36.03 (14.75), 10–65^a^	13.97 (5.86), 5–28^a^	31.77 (18.52), 5–88^b^	6.87 (3.15), 1–14^b^	*F*(3,111)=36.32, *p*<0.001
**SMFQ-c total (mean,*****SD*****, range)**	6.70 (4.50), 1–20	4.79 (3.20), 0–11	7.34 (5.77), 0–19^b^	2.17 (2.41), 0–8^b^	*F*(3,111)=8.86, *p*<0.001
**SMFQ-p total (mean,*****SD*****, range)**	6.60 (4.97), 0–21^a^	1.83 (2.28), 0–10^a^	8.63 (7.89), 0–25^b^	1.43 (1.92), 0–8^b^	*F*(3,111)=15.01, *p<*0.001

*Note*. Where self-report data was missing, this was less than 10% of the dataset. Superscript letters refer to pairwise comparisons (conducted for children with anxiety disorders versus adolescents with anxiety disorders, children with anxiety disorders versus non-anxious children, and adolescents with anxiety disorders versus non-anxious adolescents); means that share subscripts within rows are significantly different at *p*<0.05. SCAS=Spence Child Anxiety Scale, SMFQ=Short Moods and Feelings Questionnaire.

**Table 2:** Group differences in responses on the ambiguous scenarios questionnaire.

	**Anxious children (*n*=30)**	**Non-anxious children (*n*=29)**	**Anxious adolescents (*n*=30)**	**Non-anxious adolescents (*n*=30)**
**Threat (mean,*****SD*****, range)**	9.03 (4.10), 0–17	8.69 (4.11), 0–15	9.33 (4.85), 2–21^a^	4.87 (2.79), 0–12^a^
**Negative emotions (mean,*****SD*****, range)**	47.00 (18.45), 6–88	55.83 (18.65), 12–90	54.73 (24.69), 17–100	35.97 (21.03), 0–65^a^
**Perceived control (mean,*****SD*****, range)**	55.07 (21.70), 11–110^a^	52.86 (19.22), 13–92	40.17 (23.84), 2–97^a^	47.43 (25.20), 5–104
**Avoidant behavior (mean,*****SD*****, range)**	1.00 (0.95), 0–3	0.72 (1.03), 0–4	1.47 (1.43), 0–6^a^	0.67 (0.61), 0–2^a^

*Note*. superscript letters refer to pairwise comparisons (conducted for children with anxiety disorders versus adolescents with anxiety disorders, children with anxiety disorders versus non-anxious children, and adolescents with anxiety disorders versus non-anxious adolescents); means that share subscripts within rows are significantly different at *p*<0.05.

## Footnotes

1 Of the non-anxious child group, 18 cases (60%) were also included in Creswell et al.’s (2014) non-anxious sample.

## References

[bib1] Adleman N.E., Menon V., Blasey C.M., White C.D., Warsofsky I.S., Glover G.H., Reiss A.L. (2002). A developmental fMRI study of the Stroop color-word task. Neuroimage.

[bib2] Alkozei A., Cooper P.J., Creswell C. (2014). Emotional reasoning and anxiety sensitivity: associations with social anxiety disorder in childhood. J. Affect. Disord..

[bib3] Amir N., Beard C., Bower E. (2005). Interpretation bias and social anxiety. Cognit. Ther. Res..

[bib4] Anderson V.A., Anderson P., Northam E., Jacobs R., Catroppa C. (2001). Development of executive functions through late childhood and adolescence in an Australian sample. Dev. Neuropsychol..

[bib5] Angold A., Costello E.J., Messer S.C., Pickles A., Winder F., Silver D. (1995). Development of a short questionnaire for use in epidemiological studies of depression in children and adolescents. Int. J. Methods Psychiatr. Res..

[bib6] Barrett P.M., Rapee R.M., Dadds M.R. (1996). Family treatment of childhood anxiety: a controlled trial. J. Consult. Clin. Psychol..

[bib7] Barrett P.M., Rapee R.M., Dadds M.R., Ryan S. (1996). Family enhancement of cognitive style in anxious and aggressive children. J. Abnorm. Child Psychol..

[bib8] Beck A.T., Clark D.A. (1997). An information processing model of anxiety: Automatic and strategic processes. Behav. Res. Ther..

[bib9] Bögels S.M., Snieder N., Kindt M. (2003). Specificity of dysfunctional thinking in children with symptoms of social anxiety, separation anxiety and generalised anxiety. Behav. Change.

[bib10] Bögels S.M., Zigterman D. (2000). Dysfunctional cognitions in children with social phobia, separation anxiety disorder, and generalized anxiety disorder. J. Abnorm. Child Psychol..

[bib11] Butler G., Mathews A. (1983). Cognitive processes in anxiety. Adv. Behav. Res. Ther..

[bib12] Chorpita B.F., Albano A.M., Barlow D.H. (1996). Cognitive processing in children: relation to anxiety and family influences. J. Clin. Child Psychol..

[bib13] Chubb N.H., Fertman C.I., Ross J.L. (1997). Adolescent self-esteem and locus of control: a logitudinal study of gender and age differences. Adolescence.

[bib14] Creswell C., Murray L., Cooper P.J. (2014). Interpretation and expectation in childhood anxiety disorders: age effects and social specificity. J. Abnorm. Child Psychol..

[bib15] Creswell C., O’Connor T.G. (2006). Anxious cognitions in children: an exploration of associations and mediators. Br. J. Dev. Psychol..

[bib16] Creswell C., Schneiring C.A., Rapee R.M. (2005). Threat interpretation in anxious children and their mothers: comparison with nonclinical children and the effects of treatment. Behav. Res. Ther..

[bib17] Creswell C., Shildrick S., Field A.P. (2011). Interpretation of ambiguity in children: a prospective study of associations with anxiety and parental interpretations. J. Child Fam. Stud..

[bib18] Erikson E.H. (1968). Identity: Youth and Crisis.

[bib19] Essau C.A., Gabbidon J., Essau C.A., Ollendick T.H. (2013). Epidemiology, comorbidity and mental health service utilization. The Wiley-Blackwell Handbook of the Treatment of Childhood and Adolescent Anxiety.

[bib20] Foa E.B., Kozak M.J. (1986). Emotional processing of fear: exposure to corrective information. Psychol. Bull..

[bib21] Gogtay N., Giedd J.N., Lusk L., Hayashi K.M., Greenstein D., Vaituzis A.C., Toga A.W. (2004). Dynamic mapping of human cortical development during childhood through early adulthood. Proc. Natl. Acad. Sci. USA.

[bib22] Gray J.A., McNaughton N. (2003). The Neuropsychology of Anxiety: An Enquiry into the Function of the Septo-hippocampal System.

[bib23] Hooper C.J., Luciana M., Conklin H.M., Yarger R.S. (2004). Adolescents’ performance on the Iowa gambling task: implications for the development of decision making and ventromedial prefrontal cortex. Dev. Psychol..

[bib24] Hudson J.L., Rapee R.M., Deveney C., Schniering C.A., Lyneham H.J., Bovopoulos N. (2009). Cognitive-behavioral treatment versus an active control for children and adolescents with anxiety disorders: a randomized trial. J. Am. Acad. Child Adolesc. Psychiatry.

[bib25] Kendall P.C., Chansky T.E. (1991). Considering cognition in anxiety-disordered children. J. Anxiety Disord..

[bib26] Kendall P.C., Hedtke K.A. (2006). The Coping Cat Workbook.

[bib27] Kendall P.C., Treadwell R.H. (2007). The role of self-statements as a mediator in treatment for youth with anxiety disorders. J. Consult. Clin. Psychol..

[bib28] Lau J.Y.F., Belli S.R., Chopra R.B. (2013). Cognitive bias modification training in adolescents reduces anxiety to a psychological challenge. Clin. Child Psychol. Psychiatry.

[bib29] Lester K.J., Field A.P., Muris P. (2011). Experimental modification of interpretation bias about animal fear in young children: effects on cognition, avoidance behavior, anxiety vulnerability, and physiological responding. J. Clin. Child Adolesc. Psychol..

[bib30] Luna B., Garver K.E., Urban T.A., Lazar N.A., Sweeney J.A. (2004). Maturation of cognitive processes from late childhood to adulthood. Child Dev..

[bib31] MacLeod C., Mathews A., Tata P. (1986). Attentional bias in emotional disorders. J. Abnorm. Psychol..

[bib32] Mathews A., Mackintosh B. (2000). Induced emotional interpretation bias and anxiety. J. Abnorm. Psychol..

[bib33] Micco J.A., Choate-Summers M.L., Ehrenreich J.T., Pincus D.B., Mattis S.G. (2007). Identifying efficacious treatment components of panic control treatment for adolescents: a preliminary examination. Child Fam. Behav. Ther..

[bib34] Miers A.C., Blöte A.W., Bögels S.M., Westenberg P.M. (2008). Interpretation bias and social anxiety in adolescents. J. Anxiety Disord..

[bib35] Miers A.C., Blöte A.W., Heyne D.A., Westenberg P.M. (2014). Developmental pathways of social avoidance across adolescence: the role of social anxiety and negative cognition. J. Anxiety Disord..

[bib36] Mogg K., Mathews A., Eysenck M. (1992). Attentional bias to threat in clinical anxiety states. Cognit. Emot..

[bib37] Mowrer O. (1960). Learning Theory and Behavior.

[bib38] Murray L., Creswell C., Cooper P.J. (2009). The development of anxiety disorders in childhood: an integrative review. Psychol. Med..

[bib39] Nauta M.H., Scholing A., Rapee R.M., Abbott M., Spence S.H., Waters A. (2004). A parent-report measure of children’s anxiety: psychometric properties and comparison with child-report in a clinic and normal sample. Behav. Res. Ther..

[bib40] Nolen-Hoeksema S., Girgus J.S., Seligman M.E. (1992). Predictors and consequences of childhood depressive symptoms: a 5-year longitudinal study. J. Abnorm. Psychol..

[bib41] Öhman A., Mineka S. (2001). Fears, phobias, and preparedness: toward an evolved module of fear and fear learning. Psychol. Rev..

[bib42] Peris T.S., Compton S.N., Kendall P.C., Birmaher B., Sherrill J., March J., Piacentini J. (2014). Trajectories of change in youth anxiety during cognitive-behavior therapy. J. Consult. Clin. Psychol..

[bib43] Piaget J., Inhelder B. (1969). The Psychology of the Child.

[bib44] Rapee R.M., Lyneham H.J., Schniering C.A., Wuthrich V., Abbot M.A., Hudson J.L., Wignall A. (2006). The Cool Kids Child and Adolescent Anxiety Program Therapist Manual.

[bib45] Reuland M.M., Teachman B.A. (2014). Interpretation bias modification for youth and their parents: a novel treatment for early adolescent social anxiety. J. Anxiety Disord..

[bib46] Ronan K.R. (1996). Building a reasonable bridge in childhood anxiety assessment: a practitioner's resource guide. Cognit. Behav. Pract..

[bib47] Salemink E., Wiers R.W. (2011). Modifying threat-related interpretive bias in adolescents. J. Abnorm. Child Psychol..

[bib48] Silverman W.K., Albano A.M. (1996). The Anxiety Disorders Interview Schedule for DSM-IV – Child and Parent Versions.

[bib49] Silverman W.K., Saavedra L.M., Pina A.A. (2001). Test–retest reliability of anxiety symptoms and diagnoses with the anxiety disorders interview schedule for DSM-IV: child and parent versions. J. Am. Acad. Child Adolesc. Psychiatry.

[bib50] Spence S.H. (1998). A measure of anxiety symptoms among children. Behav. Res. Ther..

[bib51] Spence S.H., Barrett P.M., Turner C.M. (2003). Psychometric properties of the spence children's anxiety scale with young adolescents. J. Anxiety Disord..

[bib53] Taghavi M.R., Moradi A.R., Neshat-Doost H.T., Yule W., Dalgleish T. (2000). Interpretation of ambiguous emotional information in clinically anxious children and adolescents. Cognit. Emot..

[bib54] Telman M.D., Holmes E.A., Lau J.Y.F. (2013). Modifying adolescent interpretation biases through cognitive training: effects on negative affect and stress appraisals. Child Psychiatry Hum. Dev..

[bib55] Vassilopoulos S.P., Moberly N.J., Zisimatou G. (2013). Experimentally modifying interpretations for positive and negative social scenarios in children: a preliminary investigation. Behav. Cognit. Psychother..

[bib56] Waters A.M., Craske M.G., Bergman R.L., Treanor M. (2008). Behav. Res. Ther..

[bib57] Waters A.M., Wharton T.A., Zimmer-Gembeck M.J., Craske M.G. (2008). Threat-based cognitive biases in anxious children: comparison with non-anxious children before and after cognitive behavioural treatment. Behav. Res. Ther..

[bib58] Yurgelun-Todd D. (2007). Emotional and cognitive changes during adolescence. Curr. Opin. Neurobiol..

